# Graphical genotyping as a method to map *Ny*_*(o,n)sto*_ and *Gpa5* using a reference panel of tetraploid potato cultivars

**DOI:** 10.1007/s00122-016-2831-y

**Published:** 2016-11-21

**Authors:** Herman J. van Eck, Peter G. Vos, Jari P. T. Valkonen, Jan G. A. M. L. Uitdewilligen, Hellen Lensing, Nick de Vetten, Richard G. F. Visser

**Affiliations:** 1 Plant Breeding, Wageningen University and Research, P.O.Box 386, 6700 AJ Wageningen, The Netherlands; 2 Averis Seeds B.V., Valtherblokken Zuid 40, 7876 TC Valthermond, The Netherlands; 3 Plant Pathology Laboratory, Department of Agricultural Sciences, University of Helsinki, Helsinki, Finland

## Abstract

*****Key message***:**

**The method of graphical genotyping is applied to a panel of tetraploid potato cultivars to visualize haplotype sharing. The method allowed to map genes involved in virus and nematode resistance. The physical coordinates of the amount of linkage drag surrounding these genes are easily interpretable.**

**Abstract:**

Graphical genotyping is a visually attractive and easily interpretable method to represent genetic marker data. In this paper, the method is extended from diploids to a panel of tetraploid potato cultivars. Application of filters to select a subset of SNPs allows one to visualize haplotype sharing between individuals that also share a specific locus. The method is illustrated with cultivars resistant to *Potato virus Y* (PVY), while simultaneously selecting for the absence of the SNPs in susceptible clones. SNP data will then merge into an image which displays the coordinates of a distal genomic region on the northern arm of chromosome *11* where a specific haplotype is introgressed from the wild potato species *S. stoloniferum* (CPC 2093) carrying a gene (*Ny*_*(o,n)sto*_) conferring resistance to two PVY strains, PVY^O^ and PVY^NTN^. Graphical genotyping was also successful in showing the haplotypes on chromosome *12* carrying *Ry*-*f*_*sto*_, another resistance gene derived from *S. stoloniferum* conferring broad-spectrum resistance to PVY, as well as chromosome *5* haplotypes from *S. vernei,* with the *Gpa5* locus involved in resistance against *Globodera pallida* cyst nematodes. The image also shows shortening of linkage drag by meiotic recombination of the introgression segment in more recent breeding material. Identity-by-descent was found to be a requirement for using graphical genotyping, which is proposed as a non-statistical alternative method for gene discovery, as compared with genome-wide association studies. The potential and limitations of the method are discussed.

**Electronic supplementary material:**

The online version of this article (doi:10.1007/s00122-016-2831-y) contains supplementary material, which is available to authorized users.

## Introduction

The concept of graphical genotypes as a visual method to represent genetic marker data was first described by Young and Tanksley (1989). In a karyotype-style drawing, they used colours to display the mosaic structure of the chromosomes in F_2_ or backcross individuals according to the parental origin of the chromosomal regions or alleles. The transition of one colour into another displayed how chromosomes were transmitted and indicated the positions of crossovers that occurred during the meiosis of the F1 plants. Graphical genotypes are visually attractive and more easily interpretable by glance than the numerical information in a spreadsheet with offspring genotypes in columns and marker data in rows.

**Fig. 1:**
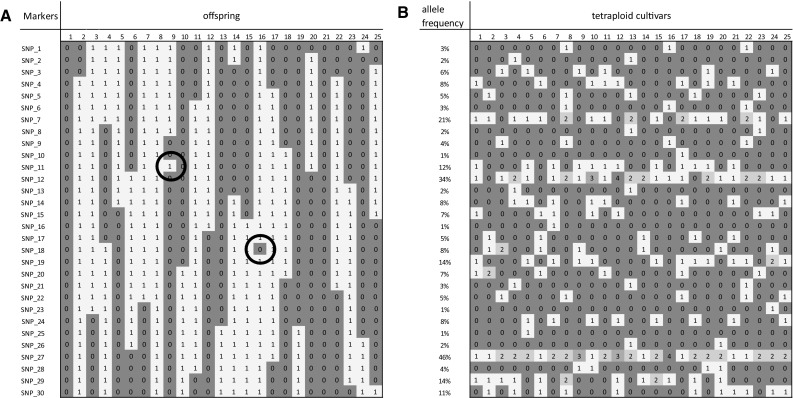
Two hypothetical examples of graphical genotypes. **a** Graphical genotypes of a bi-parental diploid backcross offspring where *colour codes* distinguish the parental origin of the chromosomal segment (*red* indicates the recurrent parent, *yellow* indicates heterozygous introgression of donor parent). Singletons, putatively indicating error data, are indicated with the *black circles*. **b** Graphical genotypes of unrelated tetraploid cultivars, where the dosage of the minor allele is indicated with numbers *0*, *1*, *2*, *3* and *4* or the shades *red*, *yellow*, *light green*, *middle green* and *dark green* (color figure online)

Graphical genotypes are used for a number of analyses. First, graphical genotypes can display the positions and the proportions of donor and recurrent genome during subsequent backcross generations (Young and Tanksley 1989; Yun et al. 2006). Second, graphical genotypes allow quick data inspection and the visual identification of singletons (Fig. 1a) that should not be interpreted as double recombinants (Van Os et al. 2005). Good data quality should result in a “Zebra striping pattern”, whereas erroneous data add isolated spots. Thirdly, high-resolution mapping of loci with major effects (Finkers-Tomczak et al. 2009) is supported by a graphical analysis of the recombinants and their trait values, allowing one to zoom into the remaining interval where candidate genes reside. Graphical genotyping is supported by software packages such as GGT (van Berloo 1999, 2008) and Flapjack (Milne et al. 2010).

Usually, graphical genotypes are generated using offspring from bi-parental crosses where two colours can encode the contribution of each of the two parents. A third colour may be needed to depict heterozygosity, but in BC_1_ or recombinant inbred lines (RILs) a third colour is not necessary. The F_1_ offspring from non-inbred parents is cumbersome, because four colours are required to show the mosaic structure in the maternally and paternally inherited recombinant chromosomes. This is easily circumvented by the separation of maternal and paternal markers and to apply graphical genotyping separately to the parental maps. An example is shown in Fig. 1a, which could represent a parental map of non-inbred parents, doubled haploids (DHs), RILs or a BC_1_.

Graphical genotyping of unrelated clones may be possible for diploid, self-pollinated species. Here, the graphical image can be used to display a local haplotype map (Motte et al. 2014) where DNA sequence variants representing either the allele of the reference genome or the non-reference allele are shown with different colour codes. Graphical genotypes and local haplotype maps are seemingly different terms (used in a mapping or haplotyping context, respectively), but they are conceptually related. Multi-colour graphical genotyping is used for depicting multiparent advanced generation inter-cross (MAGIC) lines (Bergelson and Roux 2010). Graphical genotyping of a panel of unrelated tetraploids seems to be without value, because no meaningful pattern is displayed (Fig. 1b).

*Potato virus Y* (PVY) is the most widespread and economically harmful virus infecting potatoes. It can cause 50–85% yield reduction (Valkonen 2007). PVY is transmitted by aphids and difficult to control in the field unless resistant potato cultivars are grown. Although many countries have implemented systems for the propagation of virus-free planting material, breeding for PVY resistance offers a more durable solution. Resistance genes against PVY have been introgressed in potato cultivars from many sources, as summarized in Table 1.

**Table 1:** Overview of the genetic loci involved in PVY resistance with their linkage group and ancestral germplasm

Locus name	Linkage group	Ancestral germplasm	References
*Nc*_*spl*_	4	*S. sparsipilum*	Moury et al. (2011)
*Ny*_*tbr*_	4	*S. tuberosum*	Celebi-Toprak et al. (2002)
*Ry*_*chc*_	9	*S. chacoense*	Hosaka et al. (2001); Sato et al. (2006)
*Ny*-*1*	9	cv. Rywal	Szajko et al. (2008)
*Ny*-*Smira*	9	cv. Sarpo Mira	Tomczyńska et al. (2014)
*Ry*_*adg*_	11	*S. tuberosum* Group *andigena*	Hämäläinen et al. (1997, 1998)
*Ny*-*2*	11	cv. Romula	Szajko et al. (2014)
*Ry*_*sto*_	11	*S. stoloniferum*	Brigneti et al. (1997)
*Ry*-*f*_*sto*_/*Ry*_*sto*_^a^	12	*S. stoloniferum*	Flis et al. (2005); Song et al. (2005)

^a^The locus name *Ry*_*sto*_ is used twice in the literature

*Solanum stoloniferum* is an important source of PVY resistance (Ross 1986). It carries genes for extreme resistance (ER) able to inhibit multiplication of all strains of PVY. Two genes, *Ry*-*f*_*sto*_ and *Ry*_*sto*_, for ER to PVY derived from *S. stoloniferum* have been mapped to the same region on chromosome *12* and used in variety breeding. *Ry*_*sto*_ tends to be associated with male sterility, in contrast to *Ry*-*f*_*sto*_ (Flis et al. 2005; Song and Schwarzfischer 2008). Brigneti et al. (1997) reported on the localisation of a PVY resistance from *S. stoloniferum* on chromosome *11*, but their work has not received much follow-up and their locus name *Ry*_*sto*_ has caused confusion due to synonymy with the gene subsequently described on chromosome 12. Brigneti et al. (1997) used clone I-1039, which was developed in India as cv. KHUMAL RED 2 from Scottish late blight-resistant progenitor clones and an undisclosed progenitor M 136-6. This material has *Solanum phureja* and *S. edinense* in its pedigree, but its *S. stoloniferum* origin is unclear from pedigree information presented by the authors. Clone I-1039 is rarely used in breeding and resulted in two Danish cultivars TIVOLI and LIVA, and two cultivars released in Ecuador and Rwanda, FRIPAPA 99 and GIKUNGU, respectively. Hence, it may be problematic to extrapolate the marker data of Brigneti et al. (1997), for the identification of PVY resistance beyond their experimental mapping population. Another donor of PVY resistance, clone F87084, derived from *S. stoloniferum* CPC 2093 (De Jong et al. 2001; Nie et al. 2015) has not yet resulted in cultivars released to the market and its map position is not described. Resistance to PVY originating in *S. stoloniferum* has been introgressed also via three German progenitor clones: MPI 13128 (sto × ERICA), clone 43 (sto × POLONIA), MPI 46.152/1 (sto × FRÜHMOLLE) (Song and Schwarzfischer 2008), and from the Commonwealth Potato Collection (accession CPC 2093), e.g. via the Dutch progenitor clone Y 66-13-636.

In this study, we applied graphical genotyping on unrelated tetraploid potato cultivars as a tool for mapping PVY resistance and identification of the introgressed haplotypes. The aim was to elucidate whether the Dutch potato cultivars descending from *S. stoloniferum* CPC 2093 via progenitor clone Y 66-13-636 contain a locus for resistance to PVY on chromosome *11*. Furthermore, we tested whether this method allows identification of introgression segments of the *Gpa5* locus (Rouppe van der Voort et al. 2000) and the *Ry*-*f*_*sto*_ locus (Song et al. 2005; Flis et al. 2005). Finally, we also investigated whether other traits lacking most recent common ancestor (MRCA) information can be identified using this method.

## Materials and methods

### Plant material and PVY resistance tests

A panel of 83 tetraploid cultivars and progenitor clones were included in the study (Supplementary data S1). Among these tetraploids, the authors were aware that cultivar FESTIEN is resistant to PVY, possibly due to a resistance gene derived from its great-grandparent Y 66-13-636. KARTEL, the PVY-susceptible parent of FESTIEN, was also included in the panel. Pedigree information was retrieved from our database (Berloo et al. 2007).

Validation of the PVY resistance gene was achieved by growing 180 offspring of the cross between FESTIEN (PVY resistant) × SERESTA (PVY susceptible) in pots in the greenhouse and inoculating them by hand with a PVY tuber necrosis strain (PVY^NTN^) 4 weeks after planting. Two weeks later, symptoms of primary infection in inoculated leaves were scored. All 180 genotypes were analysed by ELISA using monoclonal antiserum (obtained from Plant Research International, Wageningen, The Netherlands). The plants negative for PVY in ELISA and without symptoms were defined as resistant, whereas the remaining plants were defined as susceptible.

FESTIEN was characterized for the type of PVY resistance using two well-defined PVY strains representing the PVY strain group O (isolate PVY^O^-UK; GenBank accession no. JX424837) (Tian and Valkonen 2013) and PVY strain group N (isolate PVY^N^-NTN-Nevski; JX432967) (Tian et al. 2014). *Potato virus A* (isolate PVA-U; AJ131402) (Rajamäki et al. 1998) was also included in the experiments. The viruses were maintained in potato cv. PITO. Healthy plants of FESTIEN were multiplied by taking and rooting stem cuttings in a growth chamber under 16-h photoperiod, light intensity 250 μE m^−2^ s^−1^, temperature 23/20 °C day/night. The plants were watered as needed and fertilized with 1% fertilizer (N:P:K = 16:9:22, Yara, Espoo, Finland). Plants were side-graft-inoculated with PVY-O, PVY-N or PVA, three plants per virus, and monitored weekly for symptom development in two independent experiments. In both experiments, the uppermost fully expanded leaves were sampled for the first time at 25 and 30 days post-inoculation (dpi), respectively, monitored further for 30 and 40 days, respectively, and sampled again. The sampled leaves were tested by DAS-ELISA using polyclonal antibodies to PVY (Adgen, Auchincruive, Ayr, Scotland, UK) and monoclonal antibodies to PVA (Mab 58/0) (Rajamäki et al. 1998). Leaf samples were weighed, 1 g of tissue was ground in 3 ml of ELISA sample buffer, and two aliquots (100 µl) of the sap were transferred to two wells of the microtitre plate (Greiner Laborteknik, Frickenhausen, Germany). Leaf sap from the potato plants used as sources of virus for inoculation was included as positive controls. Colour reactions were developed using *p*-nitrophenyl phosphate as a substrate, and absorbance was recorded at 405 nm using a microtitre plate reader (Benchmark, Bio-Rad, Hercules, CA, USA). ELISA absorbance values (*A*_405_) were regarded as positive if they were two times higher (±standard deviation) than those of the healthy control plants (Fenlon and Sopp 1991).

### DNA sequence variants

The sequence of the reference genome of *Solanum tuberosum* Group Phureja DM1-3 516R44 (DM) was obtained from the Potato Genome Sequencing Consortium (PGSC 2011). The order of superscaffolds (referred to as DMB followed by the scaffold number) is according to version 4.03 and can be retrieved from the study by Sharma et al. (2013). Next-generation sequencing of a panel of 83 tetraploid cultivars allowed the identification of 129,156 DNA sequence variants (Uitdewilligen et al. 2013). Here, the relative read depth at variant positions was used for tetraploid genotype calling in the cultivars. Further details about the methods that resulted in this dataset can be retrieved from the paper by Uitdewilligen et al. (2013). A DNA sequence variant from this study may refer to a SNP, a multinucleotide polymorphism (MNP), indels, and/or multi-allelic SNPs, hereafter for brevity collectively referred to as SNPs.

Genotype calling can be performed using the following terms: the reference (DM) and alternative (non-DM) allele, or the minor and major allele, abbreviated hereafter as REF and ALT or MIN and MAJ. Here, genotype calls representing the ALT dosage values indicate that a genotype has 0, 1, 2, 3 or 4 copies of the non-DM allele as compared with the DM reference (REF) genome. The cultivar panel of 83 tetraploids represents a population of 332 alleles (4 × 83) and for each DNA variant its allele frequency has been calculated as the sum of the copies per cultivar divided by 332. Hence, population allele counts ranging from 1 to 166 result in a minor allele frequency (MAF), and the allele with such an allele frequency equal or below 50% is considered as the minor allele (MIN). Allele counts ranging from 167 to 331 result in an allele frequency >50% and such alleles are regarded as the major allele (MAJ).

The colour codes are not defined relative to the DM reference genome. If the DM reference genome has a sequence variant which is quite rare in the remainder of the gene pool then the DM reference genome represents the minor allele.

### Filtering of SNPs dosage data to construct graphical genotypes

The data were loaded in Microsoft Excel where the 83 cultivars are shown in columns and the sequence variants along with their coordinates in 129,156 rows. The rows were sorted according to chromosome, superscaffold order and coordinates on pseudomolecules version 4.03 (Sharma et al. 2013). In the header row of the spreadsheet, we inserted filters and used these as logical operators (e.g. ‘IF’, ‘AND’, or ‘NOT’ statements). Specific allele frequencies can be filtered by setting the ‘number filter’ and choosing ‘is smaller than’. To remove rows with SNPs ‘NOT’ involved in, e.g. resistance, a ‘number filter’ was set at ‘is equal to zero’. The combination of setting additional filters on multiple columns (varieties) will act as the ‘AND’ operator. Unfortunately, the ‘OR’ operator to combine criteria across columns is not offered in Excel.

To select haplotypes or chromosomal regions involved in PVY resistance, the filters were set to select SNPs with a small allele frequency ‘AND’ specific minor alleles should be present in FESTIEN and Y 66-13-636 (allele dosage ≥1). For columns representing susceptible cultivars, the filter was set at zero for the minor allele, which selects for nulliplex genotypes. Graphical genotypes were displayed in colour using conditional formats in Excel where pseudo-colours indicated allele dosage per genotype ranging from red to green for nulliplex to quadruplex genotypes, respectively.

### GWAS

The above analysis was verified with a statistical analysis. For this purpose, trait values were defined for 83 potato cultivars (trait values are resistant = 1; susceptible = 0) and a naive genome-wide association study (GWAS) was performed using GenStat 15th edition (VSN International Ltd, UK).

## Results

**Fig. 2:**
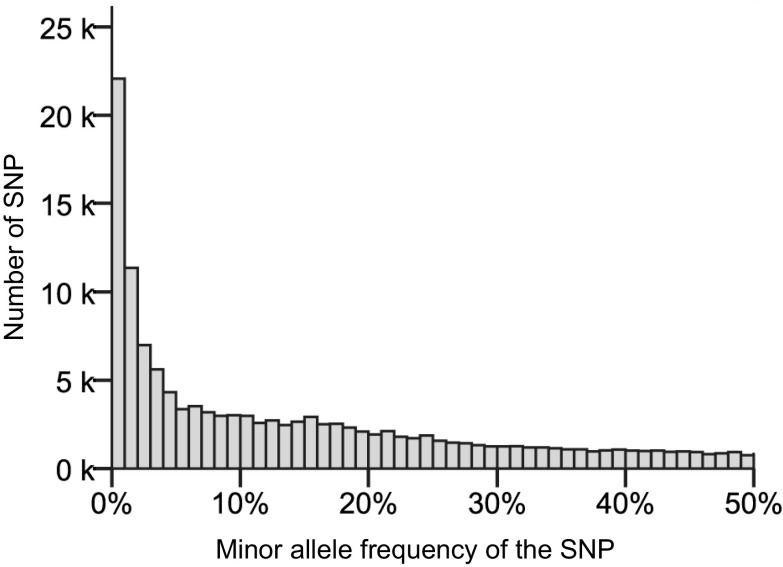
The distribution of the minor allele frequencies of all 129,156 sequence variants. The distribution indicates that many SNPs are rare variants (after Uitdewilligen et al. 2013)

When SNP data were loaded in Excel, conditional formatting according to colour scales was set as shown in Fig. 1. This resulted in a predominant background colour with rows containing fewer or greater numbers of cells having the alternative pseudo-colour according to the population allele frequency. This allows the interpretation that the vast majority of the SNPs alleles represent rare haplotypes. Figure 2 displays a clear L-shaped distribution of the allele frequencies of the SNPs. This implies that if SNPs were phased into haplotypes, the haplotype allele frequency will also display such an L-shaped distribution, which suggests that the potato gene pool is composed of many low-frequency haplotypes.

**Fig. 3:**
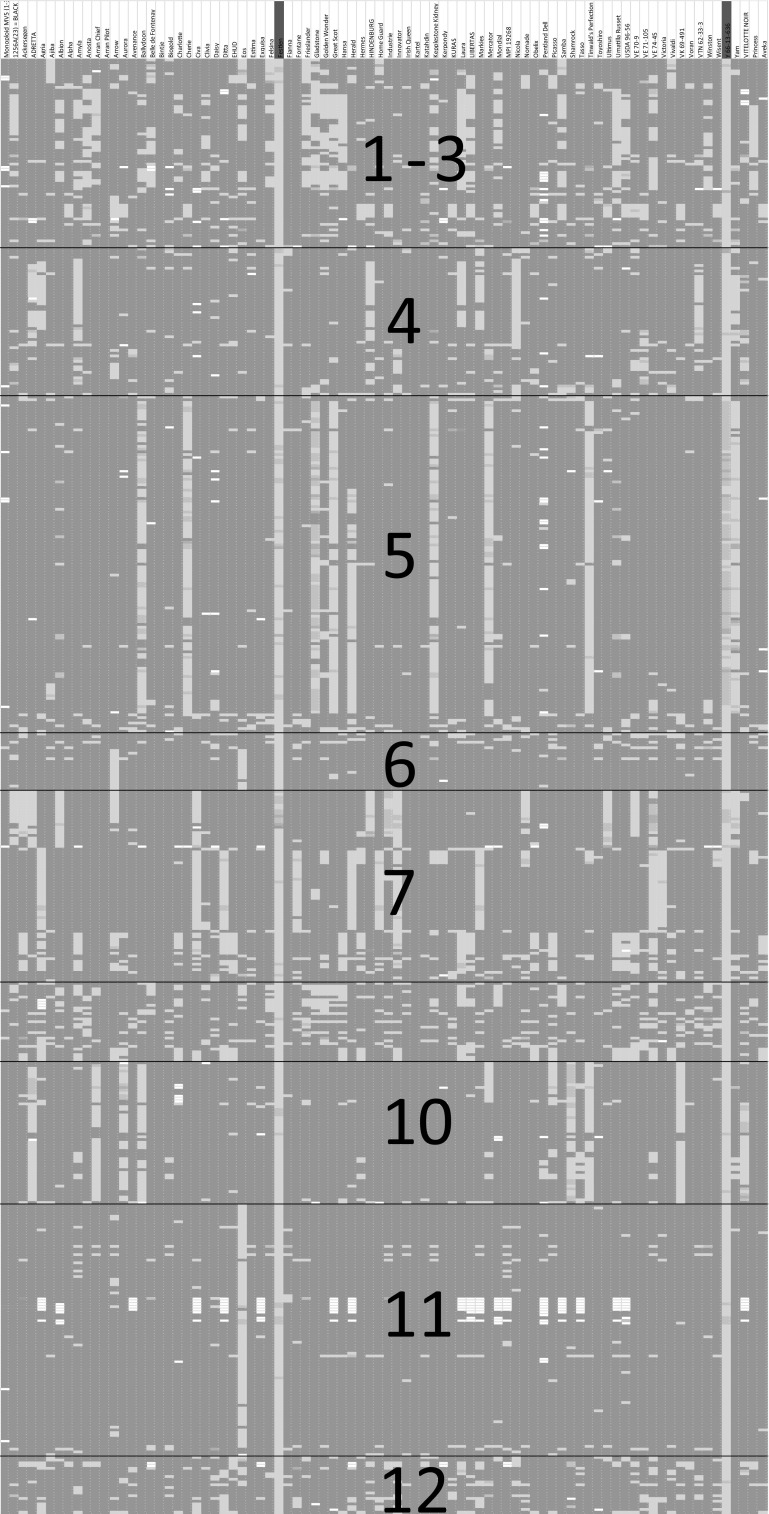
Graphical genotyping of 83 tetraploid cultivars (*columns*) with 531 SNPs (in *rows*) from 12 potato chromosomes. Absence or presence of minor alleles is indicated by conditionally formatted cells in *red* (0) or *yellow* to *green* (1, 2, 3, 4), respectively. Settings for allele dosages in FESTIEN and Y 66-13-636 were ≥1 (*yellow* to *green columns*, depending on allele dosage), whereas KARTEL, KATAHDIN, BINTJE and ARRAN PILOT were set <1 (*red columns*). The apparent pattern is indicative of haploblock sharing. The haploblock of *chromosome 11* indicates the introgression segment that carries the PVY resistance gene descending from *S. stoloniferum* CPC2093 (color figure online)

Filtering of SNPs data was performed in Excel with three criteria. As PVY resistance must have a low allele frequency, the first filter for the column with the allele count was set below 20 (allele frequency of ~6.0%). This reduced the list of 129K SNPs to 58K. The second and third filter were set to ≥1, to select for SNPs having at least ≥1 minor alleles in the column of the two PVY-resistant clones FESTIEN ‘AND’ Y 66-13-636. These clones must carry at least one allele conferring PVY resistance. The combined criteria for allele frequency ‘AND’ present in two varieties greatly reduced the number of SNPs from 58K to 748 SNPs. The next filters in the columns of PVY-susceptible clones, such as KARTEL (the susceptible parent of FESTIEN), KATAHDIN, BINTJE and ARRAN PILOT (old cultivars), were set to 0, indicating the absence of an allele involved in resistance. This selection against SNPs alleles from these four susceptible varieties resulted in only 531 SNPs matching all aforementioned criteria, which represent only 0.41% of all SNPs. Interestingly, with conditional formats these SNP data produced a specific striping pattern as shown in Fig. 3. This pattern is indeed reminiscent of graphical genotypes as observed for diploid bi-parental F_2_ or backcross populations. However, the cultivar panel is comprised of highly diverse material. If any haplotype was shared between members of the cultivar panel, then haplotype-specific SNP alleles (hs-SNPs) would merge into a yellow vertical bar in the graphical genotyping image.

Haplotype blocks could be identified for regions that belong to potato chromosome *4*, *5*, *7*, *10* and *11*. For all regions, except the region on chromosome *11*, the yellow patterns are also observed in potato cultivars that are susceptible. Based on the many false positives these regions were rejected. The candidate region on chromosome *11* indicated that one haploblock was shared by three cultivars: EOS, FESTIEN and Y 66-13-636. This prompted us to examine the phenotype and pedigree of EOS. Indeed, EOS appeared to be highly resistant against PVY, male fertile (personal communication with Jacob Eising, potato breeder at Den Hartigh B.V.), and has Y 66-13-636 as grandfather. Therefore, EOS is not a false positive but a third member of the cultivar panel with PVY resistance derived from *S. stoloniferum*.

### Size of the haploblocks

The map location and sizes of the haploblocks on potato chromosome *4*, *5*, *6*, *7*, *10* and *11* (Fig. 3) can be deduced from the physical coordinates of the first and last SNP in the block. For chromosome *4*, the haploblock is part of the peri-centromeric heterochromatin and is roughly 10 Mb long, ranging from DMB366 to DMB13 (PGSC 4.03 coordinates chr04:21464000..31110000). For chromosome *5*, the haploblock is part of the north arm and is only 1.1 Mb long, ranging from DMB51 to DMB424 (PGSC 4.03 coordinates chr05:4250427..5369289). For chromosome *6*, the haploblock with 12 SNPs, locates on the distal end of the south arm in scaffold DMB686 chr06:57791621..57992915. For chromosome *7*, the SNPs were found on two scaffolds, both on the north (short) arm. One block is part of the 2.2 Mb most distal scaffold DMB47 and one block is 8 Mb further on the 0.2 Mb scaffold DMB684. For chromosome *10*, the 1 Mb haploblock on the north arm is part of two adjacent scaffolds DMB599 and DMB338 (PGSC 4.03 coordinates chr10:3903000..4917000). For chromosome *11*, all SNPs of the haploblock belong to the most distal scaffold DMB148 on the north arm (PGSC 4.03 coordinates chr11:1..1439384). The first and the last SNP of the haploblock are 500 kb apart having coordinates chr11:284162 and chr:814554, respectively. This is approximately 500 kb from the well-known *R* gene cluster that includes genes homologous to *N* and *N*-like (*Nl*-*25*) TMV resistance genes and analogues (Hehl et al. 1999).

### The haploblock comprising PVY resistance from *S. stoloniferum* CPC 2093

Further refinement of the chromosome *11* haploblock (Fig. 3) comprising PVY resistance from *S. stoloniferum* CPC 2093 was performed to identify accurate hs-SNPs using more strict filter settings, where the allele count was set to ≤4 (allowing at most one genotyping error), and EOS, FESTIEN and Y 66-13-636 have an allele count ≥1. This resulted in a total of 65 SNPs (Supplementary data 2) which displayed the minor allele in simplex condition in EOS, FESTIEN and Y 66-13-636 and was nulliplex in the other members of the cultivar panel. These SNPs should allow marker-assisted selection in any genetic background without the risk of false-positive results. From these 65 SNPs, a subset of seven SNPs was selected for validation. Their identity and coordinates are as follows: PotVar0063974 (Chr11:284168) a [T/C] SNP, PotVar0064044 (Chr11:398248) a [T/C] SNP, PotVar0064080 (Chr11:398597..398598) a dinucleotide polymorphism [TT/GA], PotVar0064400 (Chr11:786626) a [G/A] SNP, PotVar0064470 (Chr11:787325) a [C/A] SNP, PotVar0064502 (Chr11:787571) a [C/T] SNP, and PotVar0064578 (Chr11:809990) a [T/C] SNP, all from DMB148. The mapping of PVY resistance to DMB148 suggests that the *R* gene is a member of a well-known *R* gene cluster, which was named cluster XIa-TNL in bin4-8 (Bakker et al. 2011), or cluster 49 (Jupe et al. 2012). The seven SNPs were selected to be free (as much as possible) from flanking SNPs to avoid assay failure, and were included in a custom-made 20K Infinium SNP array (Vos et al. 2015), used to analyse a much wider panel of 537 potato cultivars described by D’hoop et al. (2014). In this panel, the seven SNPs were validated, because all SNPs identified other descendants from Y 66-13-636: CUPIDO, CYCLOON, LADY CHRISTL, LADY FELICIA, MELODY, MUSICA, ORCHESTRA, ORIANA, SAVIOLA, SANTÉ and W 72-22-496, as well as MIRAKEL, descending from Y 62-2-221 (the resistant parent of Y 66-13-636). These cultivars are simplex for the minor allele and their PVY resistance is in agreement with phenotypic data from breeders’ websites. In susceptible clones, however, the SNPs identified only the major reference allele.

The Infinium array also enabled the identification of four cultivars: ARIZONA, BELANA, OSIRA, and SAGITTA which are positive for this introgression segment, but bear an unknown genetic relationship to CPC 2093. Hence, we predict that these cultivars are resistant, which was verified at the breeders’ website. Furthermore, ALTUS, AXION, CYRANO, DONALD, SERESTA and XANTIA were negative for the SNPs, although these cultivars descend from CPC2093 (Supplementary data 3). It is concluded that these clones no longer carry the introgression segment and are most likely PVY susceptible.

### Validation of the map position and phenotypic characterisation of Ny_(o,n)sto_

The locus involved in PVY resistance as identified using a panel of distantly related cultivars was validated with a new bi-parental mapping population descending from FESTIEN × SERESTA. In this mapping population, 81 individuals were found to be susceptible, whereas 98 were resistant (no symptoms, and no virus detected by ELISA, Supplementary data 4). This is in agreement with a 1:1 segregation ratio and in accordance with the Mendelian expectations of a simplex allele. The two [T/C] SNP markers PotVar63973 (Chr11:284162) and PotVar63974 (Chr11: 284168) were converted into one SNP assay and analysed for co-segregation with the PVY resistance. The SNP assay nicely predicted the PVY resistance phenotype in the offspring of 179 individuals, except for four resistant individuals. This may indicate recombination between the *R* gene and the marker locus PotVar63973/63974 in four progeny plants, but the lack of infection might also be due to unsuccessful inoculation. The latter is more likely because in the wider panel of 537 cultivars tested with the Infinium array no recombination events were observed among the SNP loci. These results provide further evidence for the localisation of the PVY resistance gene at a distal position of potato chromosome 11.

**Fig. 4:**
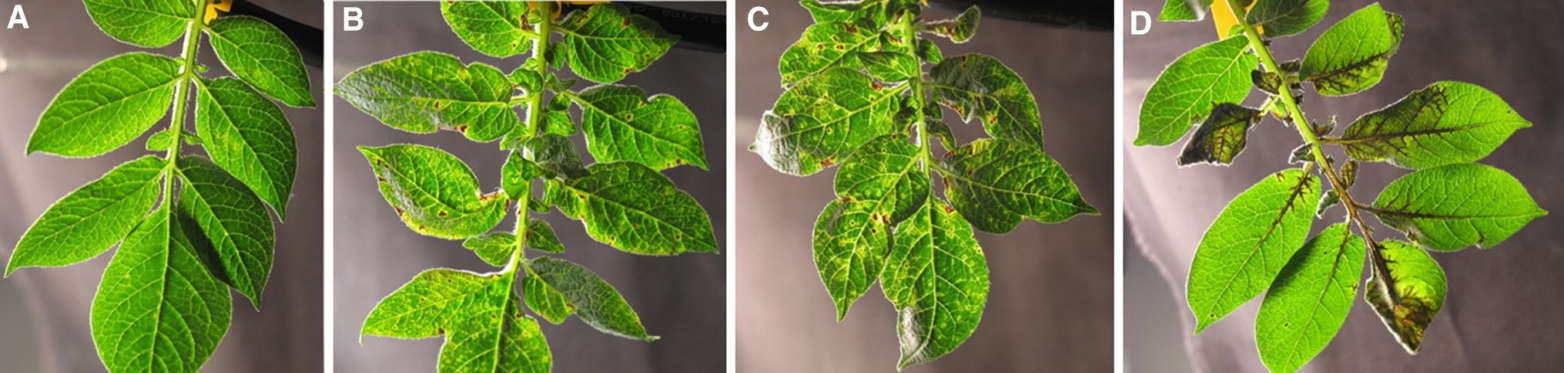
Symptoms caused by systemic infection with *Potato virus Y* (PVY) or *Potato virus A* (PVA) in the top leaves of potato cv. FESTIEN 3 weeks after side-graft inoculation of the plants. **a** A healthy leaf from a non-inoculated control; **b**, **c**, Necrotic pinpoint lesions caused by the strain PVY^NTN^-Nevski and PVY^O^-UK, respectively. **d** Rapidly expanding necrosis in the leaves systemically infected with PVA

Further characterisation of PVY resistance in FESTIEN was carried out by graft inoculation with PVY^O^-UK and PVY^N^-NTN-Nevski. Both PVY strains induced similar symptoms of necrotic pinpoint lesions in the uppermost leaves 3 weeks post graft inoculation. Necrosis advanced by time and affected also the small veins. Hence, the top leaves became heavily diseased by 6 weeks post inoculation, but they did not die. The plant continued to develop new leaves and these leaves developed similar symptoms (Fig. 4). The plants were stunted, as the top leaves did not develop normally. FESTIEN was also tested for resistance to PVA by graft inoculation, which caused severe systemic necrosis, typical of the hypersensitive resistance response (HR). Initially, veinal necrosis was observed in the top leaves ca. 3 weeks post graft inoculation, followed by development of large necrotic lesions and, eventually, death of the leaf (Fig. 4). Both PVY strains and PVA could be detected in the symptomatic leaves by DAS-ELISA, but virus concentrations in FESTIEN were at least 25–100-fold lower (1–4%) than in the susceptible cultivar PITO. Because the viruses were readily detectable by ELISA in the symptomatic leaves, albeit at very low concentration, FESTIEN was concluded to express HR to both strains of PVY and to PVA. Based on the HR phenotype, we propose *Ny*_*(o,n)sto*_ as name for this locus.

### Results of GWAS

**Fig. 5:**
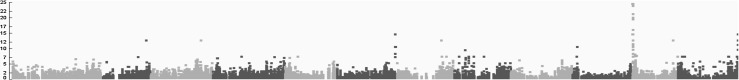
Manhattan plot of a GWAS, showing the statistical validation of the graphical mapping of *Ny*_*(o,n)sto*_ on superscaffold DMB148 on potato chromosome *11*. A secondary peak on *chromosome 12* is indicative of *Ry*-*f*_*sto*_. On the *y*-axis the −log_10_(*P*) value, and on the *x*-axis the physical positions of the SNP is shown in *alternating colours* for 12 consecutive potato chromosomes

A genome-wide association study was performed as a statistical alternative to the graphical genotyping method to analyse the significance of the mapping and the power of GWAS to identify the PVY resistance based on only four resistant cultivars (EOS, FESTIEN, KURAS and Y 66-13-636) in the panel of 83 tetraploids. We included cv. KURAS as the fourth PVY-resistant clone to mimic the situation that GWAS is based on phenotypic data and does not have a priori assumptions on trait heterogeneity (=Variation caused by different genes can give rise to the same phenotype). Figure 5 shows a Manhattan plot with the outcome of the GWAS for Y-virus resistance. A major peak can be observed for SNPs that reside in superscaffold DMB148 on chromosome *11*, matching with the coordinates of hs-SNPs in the graphical genotypes. Figure 5 illustrates that a haplotype tagged by several hs-SNPs creates a pileup in the Manhattan plot, whereas spurious or singleton SNPs were detected on chromosomes *2*, *3*, *6*, *7* and *12*. The SNPs underlying the peak on the right end of chromosome *12* reside in superscaffold DMB114 and represent the haplotype that carries *Ry*-*f*_*sto*_ (Song and Schwarzfischer 2008). The peak on the right end of chromosome *6* represents a false-positive QTL, based on the coincidental presence of two partial and two complete haplotype blocks in four varieties ARROW, EOS, FESTIEN and Y66-13-636 (see the graphical genotyping image Fig. 3), where ARROW is not descending from CPC 2093. In a GWAS, where KURAS is excluded from the analysis, the secondary peak on chromosome 12 is absent (data not shown).

### Graphical genotyping of Ry-f_sto_

We tested if graphical genotyping could also identify the *Ry*-*f*_*sto*_ locus. In our cultivar panel, the variety KURAS was the only genotype identified as being PVY resistant due to *Ry*-*f*_*sto*_. A value for allele count was set <2, and a value for a minor allele ≥1 for KURAS. This results in the identification of each of the 479 KURAS-specific SNPs as potentially associated with the *Ry*-*f*_*sto*_ haplotype. However, these SNPs were located on segments spanning the entire genome. Scaffold DMB127 on chromosome *3* contributed 75 SNPs (16%); twelve scaffolds of chromosome 7 contributed 321 SNPs (67%), and 50 SNPs (10%) localized to chromosome 12. Within chromosome 12 the SNPs localized at three positions: 11 SNPs telomeric north arm, 13 SNPs close to the centromere and telomeric south we observed 2 SNPs in DMB38 and adjacently 24 SNPs in DMB114 (length 1.6 Mb, coordinates chr12:58927871..60583523). This is indeed the *R* gene cluster tagged by STM0003 (coordinates: chr12:60055231..60055365; Song et al. 2005) and the PCR markers YES3-3A (genbank accession BV725480; BLAST hit on coordinates: chr12:59061649..59061903).

Nevertheless, this result is incorrect. Three of the SNPs from this introgression segment were present on our 20K-SNP array, but were negative for AMADO, a PVY-resistant descendant of KURAS that was present in the wider panel of 537 potato cultivars. However, other SNPs on the Infinium array could accurately predict PVY resistance based on *Ry*-*f*_*sto*_. Therefore, graphical genotyping was performed with more relaxed settings. When the filter for allele count was set ≤5 an unexpected haplotype sharing between cv. KURAS and cv. HINDENBURG could be observed in the graphical genotyping image. From other data, we already knew that our DNA was isolated from a clone incorrectly labelled as HINDENBURG.

A second attempt included this incorrectly labelled clone (presumably carrying *Ry*-*f*_*sto*_) along with the additional knowledge on the location of *Ry*-*f*_*sto*_ on DMB114, chromosome *12*. This resulted in the identification of 62 SNPs (Supplementary data 5). Four of these 62 SNPs (PotVar0052353 at DMB114:626106, PotVar0052707 at DMB114:902759, PotVar0053235 at DMB114:1376622 and PotVar0053451 at DMB114:1599751) were present on the 20K Infinium array and all four SNPs positively identified KURAS and AMADO and were negative for the remainder of the 537 cultivar panel.

### The *Solanum vernei* introgression segment with Gpa5

Another example of the application of graphical genotyping relates to the *Gpa5*_*vrn*_ locus on chromosome *5* conferring *Globodera pallida* nematode resistance, initially described using the diploid clone 3704-76 (Rouppe van der Voort et al. 2000). The resistant parental clones 3778-16 and 3704-76 are dihaploids extracted from the tetraploid progenitor clones AM 78-3778 and AM 78-3704, respectively. Both clones may carry multiple *S.vernei*-derived alleles from both the maternal and paternal side. The pedigrees of both AM 78-3778 and AM 78-3704 display the same three sources of *S. vernei* (Supplementary data 6). The first is the breeding clone LGU 8 (developed by R.L. Plaisted, Cornell University, USA) using unnamed *S. vernei* material from Hans Ross (MPIZ, Germany). The second is clone V 24/20 which points back to Scottish work using CPC 2488-3 × CPC 2487-3. Third, VRN 1–3 represent material derived from *S. vernei* hybrid GLKS 58.1642-4 (Dellaert and Vinke 1987).

**Fig. 6:**
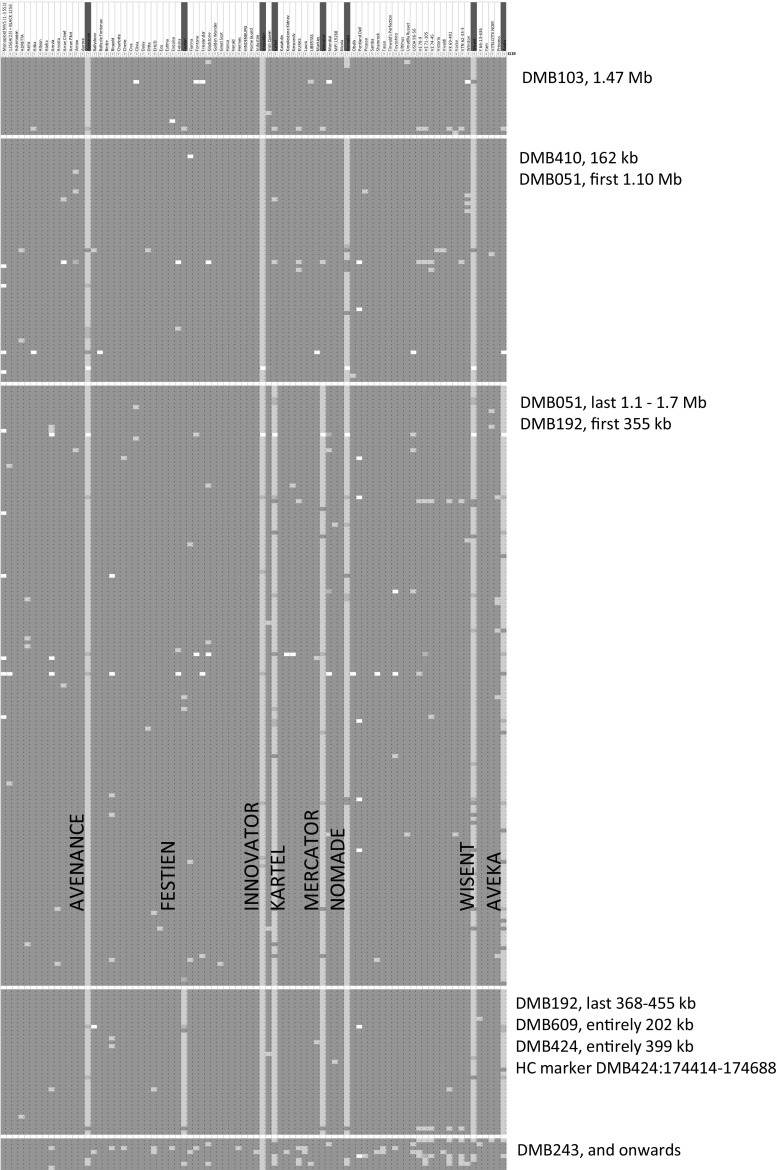
Graphical genotyping image of 83 tetraploid cultivars (*columns*) with 294 SNPs (in *rows*) from potato chromosome *5* where *Gpa5* haploblock sharing and size of linkage drag is visualized. The reference panel of 83 cultivars (*columns*) has eight cultivars with resistance against cyst nematodes *Globodera pallida* (race pa2/3). The SNPs are located on superscaffolds (DMB numbers) as defined by PGSC (2011), and specific coordinates are given in suppl. material 6. The introgressed haplotypes of AVENANCE, INNOVATOR and WISENT comprise the entire north arm of chromosome *5*. Cultivar FESTIEN has lost most linkage drag with an introgression segment of ~0.7 Mb

Cultivar INNOVATOR exhibits an exceptionally high level of resistance to the potato cyst nematode *G. pallida*, which is derived from progenitor AM 78-3778. Hence, we filtered on INNOVATOR as the resistant clone (50K SNPs), selected SNPs from chromosome 5 (5352 SNPs remain) with an allele count <20 (615 SNPs remain), and used ARRAN PILOT, BINTJE, CHARLOTTE, CIVA, DAISY, GOLDEN WONDER, HOME GUARD, KATAHDIN, ULTIMUS and YAM as negatives for *G. pallida* resistance. This resulted in 294 remaining SNPs according to the pattern shown in Fig. 6.

The graphical genotyping image shown in Fig. 6 shows a potentially erroneous orientation of DMB103. A reorientation of DMB103 would nullify the gap.

The transitions from green to red in the columns of Fig. 6 also display the positions of the historical recombination events, which caused a reduction of the linkage drag associated with *Gpa5* introgression. The cultivars AVENANCE (2005), INNOVATOR (1999) and WISENT (2005) have the largest introgression segment, followed by NOMADE (1995). In MERCATOR (1999) and KARTEL (1994), the introgression segment is again shortened and linkage drag is minimal in FESTIEN (2000). The shortening of the introgression segment (which is the same as the removal of linkage drag) does not follow a trend from older to more recently released cultivars. Therefore, the historical recombination events must have taken place in between generations deeper in the pedigrees.

The shortest segment observed in cv. FESTIEN is consistent with the location of the HC marker (Achenbach et al. 2009). The HC marker is known to produce the least amount of false negatives of all markers developed to select *Gpa5*. To compare the physical coordinates of the HC marker relative to the graphical location of *Gpa5* (Fig. 6), a BLAST search was performed on the pseudomolecules of PGSC v4.03, using the primer sequences of the HC marker. BLAST indeed finds the best hit within DMB424 at superscaffold coordinates 174417..174688.

### Negative results

Unfortunately, we were unable to find SNPs indicating the *Gro1*–*4* gene on chromosome *7* involved in resistance against *Globodera rostochiensis* nematodes (also known as the *Fb* gene, Paal et al. 2004). This effort failed because it is unknown how the experimental material, from which the gene was cloned, is related to cultivars that entered the market.

## Discussion

Intuitively one may easily accept that application of a few selection criteria imposed on a data set with 129,156 SNPs allows the graphical visualization of haploblocks. To explain the underlying principles is, however, not as easy. SNPs are binary characters which allow one to separate haplotypes into two groups: a group with haplotypes that share the SNP allele and a group of haplotypes that share the alternative variant. The number of haplotypes in the potato gene pool is, however, much larger and can be deduced from the large number of SNPs with a low minor allele frequency (MAF) as shown in Fig. 2. The average MAF is 0.14 only. Moreover, 17.4% of all SNPs have a MAF <1%, and 39.1% have a MAF <5% (Uitdewilligen et al. 2013). Supposing that all SNPs with an MAF <5% are haplotype specific (hs-SNPs), this allows one to imagine that the gene pool is comprised of at least a dozen different haplotypes per locus. Apart from the hs-SNPs, the remaining SNPs with a higher MAF may consign haplotypes into two groups, without any reason to assume that the alleles within such a group have anything in common.

Graphical genotyping thus relies on the ability to distinguish specific minor alleles because of the presence of hs-SNPs against the many other haplotypes which share the absence of this minor hs-SNP allele. In other words, haplotype specificity refers to the minor allele. The major allele is not haplotype specific at all.

A negative result of graphical genotyping was the inability to find SNPs indicating the *Gro1*–*4* locus. Possibly the *Gro1*–*4* gene is hardly deployed in variety breeding and, therefore, escapes detection in this limited variety panel. Furthermore, *Ro5*-resistant cultivars descending from *S. spegazzinii* have an overly complex pedigree resulting in ambiguity if they share any most recent common ancestor. We also obtained negative results for several other single locus traits, such as flesh colour (Wolters et al. 2010), tuber shape (Van Eck et al. 1994) and maturity (Kloosterman et al. 2013). This is likely to be due to a violation of the assumption of identity-by-descent. If a phenotype is not the result of a single (recent) mutation, also indicated as genetic heterogeneity, then other (statistical) methods will also fail. In addition, the size of the haploblock may be too short to allow the identification of a meaningful tract of SNPs in a graphical genotyping image. Finally, it should be noted that although haplotypes are defined by SNPs, it is not necessarily that any of these SNPs are haplotype specific. We, therefore, conclude that the power of graphical genotyping and the power of statistical methods equally suffer from trait heterogeneity and genetic heterogeneity.

Another notion to understand this application of graphical genotyping in unrelated tetraploids is based on the breeding history of potato cultivars. During a century of potato breeding and approximately one meiosis per decade (Love 1999), large haploblocks can be expected. The gene pool has a limited number of founders and donors of resistance and, therefore, identity-by-descent is obvious for haplotypes shared by related cultivars. These aspects contribute to long haploblocks. Shorter haploblocks associated with traits can also appear by chance. The probability for such random effects is also influenced by the nucleotide diversity between haplotypes.

Finally, within a given level of LD, nucleotide diversity and founders, the number of SNPs needs to exceed a certain minimal number. Only with a large number of SNPs clear stripes will appear within the image, where the length of the stripes enables the visual identification of haplotype sharing. Future work may show that the number of markers comprised by each stripe is indicative of the statistical significance for the conclusion of haplotype sharing or IBD. In Table 2, a summary is given of the many aspects that contribute to the success or failure of graphical genotyping. Most aspects have a similar impact on the success of genome-wide association studies (GWAS).

**Table 2:** Overview of aspects that contribute to the success or failure of graphical genotyping

Aspect	Impact on the success of graphical genotyping	Impact on statistical aspects of association analysis
Identity-by-descent	IBD is a prerequisite for graphical genotyping, whereas genetic heterogeneity is fatal	Genetic heterogeneity is detrimental for the statistical power in association analysis
SNP density	Large numbers of SNPs will merge into longer tracts. This facilitates visual recognition of haplotype size and haplotype sharing from GGT images	Association analysis evaluates the statistical significance of individual SNPs, whereas the number and distances of nearby supporting SNPs is only considered occasionally upon closer inspection of the Manhattan plot
LD	Long range LD will result in larger visual tracts at a given SNP density	Decay of LD is detrimental in association analysis
Nucleotide diversity	Higher diversity gives more SNPs and more hs-SNPs. Identification of haplotype sharing is based on the amount of hs-SNPs	hs-SNPs will have higher statistical power in association studies
Gene pool size and founders	A wider genetic basis and introgression facilitates the ability to differentiate haplotypes. The number of haplotypes is proportional to the number of founding fathers	Statistical power is negatively influenced by a higher number of haplotypes or low SNP allele frequencies
Haplotype size	Graphical genotyping can show the exact length and coordinates of the haplotypes and progress in removal of linkage drag	GWAS does not display the size of haplotypes
Monogenic or polygenic traits	Graphical genotyping is most easily applied to monogenic characters	Trait heterogeneity is detrimental for GWAS, but polygenic traits can be dissected

Most aspects have a similar impact on the success of genome-wide association studies (GWAS)

We have mapped a locus involved in PVY resistance derived from CPC 2093. The putative resistance gene in the locus was tentatively named *Ny*_*(o,n)sto*_ following guidelines (Valkonen et al. 1996), because the resistant parent (FESTIEN) of the gene mapping population expressed HR to both PVY^O^ and PVY^NTN^. However, the mapping population was studied only for segregation of resistance to PVY^NTN^ and remains to be tested with PVY^O^ before involvement of the same locus in resistance to both PVY strains can be confirmed. Furthermore, we showed that FESTIEN responds with HR to PVA. Co-segregation of resistance to PVY and PVA is known in *S. stoloniferum* (Cockerham 1970), but co-segregation of the gene markers and HR to PVA should be tested to identify the PVA resistance locus.

In view of HR conferred by *Ny*_*(o,n)sto*_ and ER conferred by *Ry*_*sto*_ and *Ry*-*f*_*sto*_ (Brigneti et al. 1997; Flis et al. 2005; Song et al. 2005; Valkonen et al. 2008), the genes underlying these resistances should be different. Intriguingly, the PVY-resistant variety TIVOLI, a first-generation descendant of the resistant parent I 1039, which was used by Brigneti et al. (1997), was positive for the SNP marker from this paper according to marker assays tested at HZPC (personal communication). A second discrepancy between marker data, pedigree information and resistance phenotype that is relevant in the context of possible shared identity with *Ny*_*(o,n)sto*_ relates to the *Ny*-*2* locus (Szajko et al. 2014). The *Ny*-*2* locus mapped in the German variety ROMULA relased in 2002. ROMULA descends from VE 79-113, which descends via W 72-38-720 from Y 66-13-636). ROMULA was characterized to confer HR to PVY, whereas SANTÉ (an F_1_ from Y 66-13-636) showed ER. Both ROMULA and SANTÉ have identical marker scores and these should be identical by descend according to their pedigrees. Not only Szajko et al. (2014) but also Heldák et al. (2007) classified SANTÉ as ER based on low ELISA readings. When judging from the ROMULA perspective, the *Ny*_*(o,n)sto*_ and *Ny*-*2* locus could be identical, but this is at odds with the phenotypic classification of SANTÉ being ER. These discrepancies may be explained by two mechanisms. First, the genes (*Ry*) for ER to PVY are epistatic to the genes (*Ny*) for HR and, hence, only the resistance phenotype conferred by *Ry* can be observed in the presence of both types of genes in the same plant (Valkonen et al. 1994). Second, the role of *Ry* and *Ny* is to recognize the virus, which triggers the signalling cascades for effective defence responses. However, allelic variation in the genes involved in signalling for defence, or in the defence mechanism itself, may render the defence less effective, which results in an altered phenotypic outcome. For example, detectable accumulation of PVY is usually prevented in potato plants carrying *Ry*, no matter whether plants are sap-inoculated or graft-inoculated (Valkonen et al. 1994; Hämäläinen et al. 1997). Nevertheless, some potato genotypes carrying *Ry* may contain low but detectable PVY titres and systemic infection following graft inoculation, which results in small necrotic lesions and/or veinal necrosis in the top leaves. While *Ny* normally prevents movement of PVY^O^ from the inoculated leaves to other parts of the plant, genetic variability in the defence signalling cascades may cause temperature sensitivity, which allows systemic infection at elevated temperatures (Valkonen 1997; Szajko et al. 2014). Wider testing and comparative analysis with, e.g. marker B11.6 (Szajko et al. 2014) is required.

Our SNP markers matching the haplotype shared by EOS, FESTIEN and Y 66-13-636 allow marker-assisted breeding. Subsequent validation with an Infinium SNP array on a wider panel of 537 cultivars (Vos et al. 2015) could identify PVY-resistant or -susceptible varieties without any discrepancy. We conclude that graphical genotyping is not only suitable to map loci in bi-parental mapping populations, but also in panels of distantly related cultivars. The graphical genotyping patterns observed here suggest the presence of specific haplotypes which are uniquely tagged by tracts of haplotype-specific SNPs.

Graphical genotyping was initially proposed as a tool in mapping studies, but it also makes singleton observations visible to allow correction of erroneous genotyping results. Whereas the data structure from a panel of cultivars (as shown in Fig. 1b) suggests that correction of genotyping errors or the identification of mislabelled of plant material is not straightforward, this paper shows that skilful use of graphical genotyping images in cultivar panels can assist in making various interpretations of the data, including observation of trait heterogeneity and genetic heterogeneity.

### **Author contribution statement**

Conceived and designed the experiments: HvE. Performed the experiments: JU, HL, NdV, JV. Analysed the data: HvE, JU, PV, JV. Wrote the paper: HvE, PV, JV.

## Electronic supplementary material

Below is the link to the electronic supplementary material.

Supplementary material 1 (DOCX 18 kb)

Supplementary material 2 (XLSX 54 kb)

Supplementary material 3 (DOCX 20 kb)

Supplementary material 4 (DOCX 94 kb)

Supplementary material 5 (XLSX 47 kb)

Supplementary material 6 (XLSX 112 kb)
