# Supplementary material for: Graphical genotyping as a method to map Ny(o,n)sto and Gpa5 using a reference panel of tetraploid potato cultivars
Source: Theor Appl Genet. 2016 Nov 21;130(3):515–28. doi: 10.1007/s00122-016-2831-y (PMC5315735; doi:10.1007/s00122-016-2831-y)
Supplement: Supplementary file 1 — Supplementary material 1 (DOCX 18 kb) [file 122_2016_2831_MOESM1_ESM.docx]

Supplementary material 1

**Graphical genotyping as a method to map *Ny_(o,n)sto_* and *Gpa5* using a reference panel of tetraploid potato cultivars.**

Herman J. van Eck*, Peter G. Vos, Jari P.T. Valkonen, Jan G.A.M.L. Uitdewilligen, Hellen Lensing, Nick de Vetten, Richard G.F. Visser

*corresponding author, e-mail address: [herman.vaneck@wur.nl](mailto:herman.vaneck@wur.nl), Wageningen University, P.O.Box 386, 6700 AJ Wageningen, The Netherlands

Panel of 83 tetraploid potato cultivars and progenitor clones, their year of (market) release and the country of origin

1256A(23) = BLACK 1256 1945 GB

Ackersegen 1929 GER

ADRETTA 1975 GER

Agria 1985 GER

Ajiba 1992 HOL

Albion 1895 HOL

Alpha 1925 HOL

Amyla 1999 FRA

Anosta 1975 HOL

Arran Chief 1911 GB

Arran Pilot 1930 GB

Arrow 2004 HOL

Aurora 1972 HOL

Avenance 2005 HOL

Ballydoon 1931 GB

Belle de Fontenay 1885 FRA

Bintje 1910 HOL

Biogold 2004 HOL

Charlotte 1981 FRA

Cherie 1997 FRA

Civa 1960 HOL

Clivia 1962 GER

Daisy 1998 FRA

Ditta 1989 AUT

EHUD 1965 HOL

Eos 2000 HOL

Estima 1973 HOL

Exquisa 1992 GER

Felsina 1992 HOL

Festien 2000 HOL

Fianna 1987 HOL

Fontane 1999 HOL

Frieslander 1990 HOL

Gladstone 1932 GB

Golden Wonder 1906 GB

Great Scot 1909 GB

Hansa 1957 GER

Herald 1928 GB

Hermes 1973 AUT

HINDENBURG 1916 GER

Home Guard 1943 GB

Industrie 1900 GER

Innovator 1999 HOL

Irish Queen 1900 GB

Kartel 1994 HOL

Katahdin 1932 USA

Kepplestone Kidney 1900 GB

Kerpondy 1949 FRA

KURAS 1996 HOL

Laura 1998 GER

LIBERTAS 1946 HOL

Markies 1997 HOL

Mercator 1999 HOL

Mondial 1987 HOL

MPI 19268 1945 GER

Nicola 1973 GER

Nomade 1995 HOL

Obelix 1988 HOL

Pentland Dell 1961 GB

Picasso 1994 HOL

Samba 1989 FRA

Shamrock 1900 IRL

Tasso 1963 GER

Tinwald's Perfection 1914 GB

Toyoshiro 1976 JPN

Ultimus 1935 HOL

Umatilla Russet 1998 USA

USDA 96-56 1945 USA

VE 70-9 1970 HOL

VE 71-105 1971 HOL

VE 74-45 1974 HOL

Victoria 1997 HOL

Vivaldi 1998 HOL

VK 69-491 1969 HOL

Voran 1931 GER

VTN 62-33-3 1962 HOL

Winston 1992 GB

Wisent 2005 HOL

Y 66-13-636 1966 HOL

Yam 1787 GB

VITELOTTE NOIR 1815 FRA

Princess 1998 BRD

Aveka 2001 HOL
