# Supplementary material for: Graphical genotyping as a method to map Ny(o,n)sto and Gpa5 using a reference panel of tetraploid potato cultivars
Source: Theor Appl Genet. 2016 Nov 21;130(3):515–28. doi: 10.1007/s00122-016-2831-y (PMC5315735; doi:10.1007/s00122-016-2831-y)
Supplement: Supplementary file 3 — Supplementary material 3 (DOCX 20 kb) [file 122_2016_2831_MOESM3_ESM.docx]

**Supplementary Material S3:** List of descendants with CPC2093 in its ancestry

**Graphical genotyping as a method to map *Ny_(o,n)sto_* and *Gpa5* using a reference panel of tetraploid potato cultivars.**

Herman J. van Eck*, Peter G. Vos, Jari P.T. Valkonen, Jan G.A.M.L. Uitdewilligen, Hellen Lensing, Nick de Vetten, Richard G.F. Visser

*corresponding author, e-mail address: herman.vaneck@wur.nl, Wageningen University, P.O.Box 386, 6700 AJ Wageningen, The Netherlands

Data from <http://www.plantbreeding.wur.nl/potatopedigree/index.html>

Van Berloo, R., Hutten, R.C.B., Van Eck, H.J., Visser, R.G.F. (2007) An online potato pedigree database resource. Potato research 50(1):45-57.

| Y 62-2-221 |  |  |  |
| --- | --- | --- | --- |
|  | VE 70-86 |  |  |
|  |  | KA 80-1596 |  |
|  |  |  | KA 87-2304 |
|  | Y 66-13-610 |  |  |
|  |  | SONATE |  |
|  |  |  | PRUMARE |
|  |  |  | SERESTA |
|  |  |  | SOPHYTRA |
|  |  |  | SOPHYTRA |
|  | Y 66-13-628 |  |  |
|  |  | VE 74-137 |  |
|  |  |  | SAMANTA |
|  |  | VE 75-209 |  |
|  |  |  | HZ 86 E 423 |
|  |  | VE 77-146 |  |
|  |  |  | SJAMERO |
|  | Y 66-13-636 |  |  |
|  |  | BALLADE |  |
|  |  | CONCORDE |  |
|  |  | DONNA |  |
|  |  | ELGAR |  |
|  |  | ELISE |  |
|  |  |  | ARTURIA |
|  |  | EVELIN |  |
|  |  | GLOBE |  |
|  |  | KONST 77-1847 |  |
|  |  |  | KONST 81-820 |
|  |  | MINERVA |  |
|  |  |  | RAPIDO |
|  |  |  | RIVIERA |
|  |  | SANTE |  |
|  |  |  | BEETS 84-85-32 |
|  |  |  | BISTRA |
|  |  |  | CELINE |
|  |  |  | FRIAR |
|  |  |  | HORIZON |
|  |  |  | NIKA |
|  |  |  | OSPREY |
|  |  |  | SAMANTA |
|  |  |  | TIFFANY |
|  |  | SUNBURY |  |
|  |  | VE 78-61 |  |
|  |  |  | PERFECTO |
|  |  | VE 80-44 |  |
|  |  |  | MARY GOLD |
|  |  |  | VECHTSTER |
|  |  | VITAL |  |
|  |  |  | HZ 87 AM 27 |
|  |  | W 72-19-443 |  |
|  |  |  | BALLYS |
|  |  |  | DONALD |
|  |  |  | PRUDENTA |
|  |  | W 72-22-489 |  |
|  |  |  | ACRONET |
|  |  |  | CEB 82-171-11 |
|  |  |  | CYCLOON |
|  |  |  | HAA 82-807-34 |
|  |  |  | OLIVIA |
|  |  |  | PARADE |
|  |  | W 72-22-496 |  |
|  |  |  | APRILIA |
|  |  |  | AR 85-102-1 |
|  |  |  | ARIADNE |
|  |  |  | BARIL |
|  |  |  | CMK 87-206-001 |
|  |  |  | CUNERA |
|  |  |  | CUPIDO |
|  |  |  | EOS |
|  |  |  | HZ 87 AM 47 |
|  |  |  | LADY FELICIA |
|  |  |  | MELODY |
|  |  |  | PALOMA |
|  |  |  | SALUD |
|  |  |  | SIROCCO |
|  |  |  | VE 82-96 |
|  |  |  | VITESSE |
|  |  |  | XANTIA |
|  |  | W 72-38-720 |  |
|  |  |  | AM 82-3167 |
|  |  |  | DOCENT |
|  |  |  | NELSON |
|  |  |  | VE 79-113 |
|  |  | WS 73-3-301 |  |
|  |  |  | BL 81-4-259 |
|  |  | WS 73-3-391 |  |
|  |  |  | LADY CHRISTL |
|  |  |  | LADY FLORINA |
|  | Y 66-18-846 |  |  |
|  |  | SOLIST |  |
|  | Y 68-4-103 |  |  |
|  |  | KONST 77-1485 |  |
|  |  |  | KONST 83-1372 |
|  |  | MINERVA |  |
|  |  | MIRAKEL |  |
|  |  |  | CMK 91-088-016 |
|  |  | MORNING GOLD |  |
|  |  | SALOME |  |
