# Supplementary material for: Graphical genotyping as a method to map Ny(o,n)sto and Gpa5 using a reference panel of tetraploid potato cultivars
Source: Theor Appl Genet. 2016 Nov 21;130(3):515–28. doi: 10.1007/s00122-016-2831-y (PMC5315735; doi:10.1007/s00122-016-2831-y)
Supplement: Supplementary file 4 — Supplementary material 4 (DOCX 94 kb) [file 122_2016_2831_MOESM4_ESM.docx]

**Supplementary material 4:** Observed phenotypes in FESTIEN × SERESTA offspring, two weeks after mechanical inoculation, with a PVY^NTN^ field isolate.

**Graphical genotyping as a method to map *Ny_(o,n)sto_* and *Gpa5* using a reference panel of tetraploid otato cultivars.**

Herman J. van Eck*, Peter G. Vos, Jari P.T. Valkonen, Jan G.A.M.L. Uitdewilligen, Hellen Lensing, Nick de Vetten, Richard G.F. Visser

*corresponding author, e-mail address: herman.vaneck@wur.nl, Wageningen University, P.O.Box 386, 6700 AJ Wageningen, The Netherlands


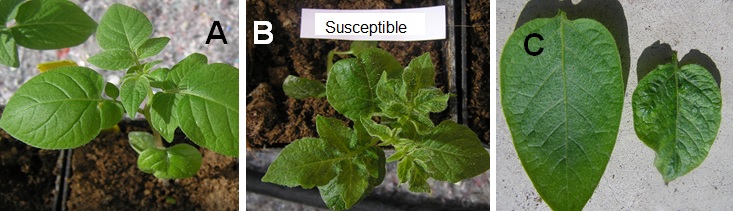


Panel A: image of a descendant conferring resistance to PVY^NTN^,

Panel B: image of a susceptible descendant, showing mosaic patterns

Panel C: image of leaves cut from a whole plant, to show the lack of a phenotype from a resistant genotype (left) and the mosaic phenotype of the susceptible descendant (right).
